# Case Report: Causative *De novo* Variants of *KCNT2* for Developmental and Epileptic Encephalopathy

**DOI:** 10.3389/fgene.2021.649556

**Published:** 2021-06-30

**Authors:** Pan Gong, Xianru Jiao, Dan Yu, Zhixian Yang

**Affiliations:** ^1^Department of Pediatrics, Peking University First Hospital, Beijing, China; ^2^Department of Pediatrics, West China Second University Hospital, Sichuan University, Chengdu, China

**Keywords:** epilepsy, *KCNT2*, genetic, seizures, encephalopathy

## Abstract

**Objective:** *KCNT2* gene mutations had been described to cause developmental and epileptic encephalopathies (DEEs). In this study, we presented the detailed clinical features and genetic analysis of two unrelated patients carrying two *de novo* variants in *KCNT2* and reviewed eight different cases available in publications.

**Methods:** Likely pathogenic variants were identified by whole exome sequencing; clinical data of the patients were retrospectively collected and analyzed.

**Results:** Our two unrelated patients were diagnosed with Ohtahara syndrome followed by infantile spasms (IS) and possibly the epilepsy of infancy with migrating focal seizures (EIMFS), respectively. They both manifested dysmorphic features with hirsute arms, thick hair, prominent eyebrows, long and thick eyelashes, a broad nasal tip, and short and smooth philtrum. In the eight patients reported previously, two was diagnosed with IS carrying a ‘change-of-function' mutation and a gain-of-function mutation, respectively, two with EIMFS-like carrying a gain-of-function mutation and a loss-of-function mutation, respectively, one with EIMFS carrying a loss-of-function mutation, three with DEE without functional analysis. Among them, two patients with gain-of-function mutations both exhibited dysmorphic features and presented epilepsy phenotype, which was similar to our patients.

**Conclusion:** Overall, the most common phenotypes associated with *KCNT2* mutation were IS and EIMFS. Epilepsy phenotype associated with gain- and loss-of-function mutations could overlap. Additional *KCNT2* cases will help to make genotype-phenotype correlations clearer.

## Introduction

Developmental and epileptic encephalopathies (DEEs) comprise a heterogeneous group of sever neurological disorders with onset in infancy and childhood, which is characterized by refractory seizures, frequent epileptic activity, and developmental regression or further slowing (Scheffer et al., 2017). Increasing number of genes identified as the cause of DEEs and channelopathies represent an important and broad class (Kumar et al., 2016). A systematic review of neurological disorders and potassium channelopathies revealed pathogenic variants in 19 potassium channel genes, including *KCNMA1, KCNN3, KCNT1, KCNT2, KCNB1, KCNJ6, KCNJ10, KCNJ11, KCNA2, KCNA4, KCND3, KCNH1, KCNQ2, KCNAB1, KCNQ3, KCNQ5, KCNC1, KCNC3*, and *KCTD3* (Kessi et al., 2020). There is a large phenotypic and genetic heterogeneity and the majority of genetic defects are still unknown. Recently, pathogenic variants in *KCNT2* gene that encodes the K_Na_1.2 subunit (Slick or Slo2.1) have been identified in eight cases (Gururaj et al., 2017; Ambrosino et al., 2018; Alagoz et al., 2020; Inuzuka et al., 2020; Mao et al., 2020). The *KCNT2*-associated DEEs comprises West syndrome, Lennox-Gastaut syndrome (LGS) as well as epilepsy of infancy with migrating focal seizures (EIMFS). *In vitro* functional analysis suggested that both gain and loss of function variants in *KCNT2* may lead to DEEs (McLachlan et al., 2019). Here, we reported two *de novo KCNT2* variants in two unrelated patients diagnosed with DEE charactering by profound developmental delay and intractable infantile-onset seizure disorders.

## Case Presentation

In total, two unrelated boys were enrolled in our study. Clinical features of affected individuals with *KCNT2* variants were summarized in Table 1.

**Table 1:** Patients with *KCNT2* variants in this publication and previous literatures.

	**This publication**		**Gururaj et al. (2017)**	**Ambrosino et al. (****2018****)**	
	**Patient 1**	**Patient 2**	**Patient 3**	**Patient 4**	**Patient 5**
Gender/Age	Male/5 m	Male/9 y 10 m	Male/4 y	Female/9 y	Female/14 y
Variant	c.991T>A, p.(Tyr331Asn)	c.592C>G, p.(Gln198Glu)	c.720T>A, p.(Phe240Leu)	c.569G>A, p.(Arg190His)	c.569G>C, p.(Arg190Pro)
Transcript	NM_198503.5	NM_198503.5	NM_001287819.1	NM_001287820.2	NM_001287820.2
Functional analysis	NA	NA	Change-of-function	Gain-of-function	Gain-of-function
Diagnosis	Ohtahara syndrome followed by IS	DEE with migrating focal seizures (EIMFS-like)	IS	IS followed by LGS	DEE with migrating focal seizures (EIMFS-like)
Age at epilepsy onset	8 d	45 d	3 m	8 m	1st day of life
Seizure type	ES	Focal and migrating seizures	Focal seizures, myoclonus, ES, tonic seizures, atypical absence	ES, nocturnal tonic and bilateral tonic-clonic seizures, non-convulsive SE	Generalized seizures, absences
Antiepileptic Treatment	PB, TPM	VPA, TPM, LTG, NZP	TPM, NZP, LEV, LTG, VBG, ESX, PLP, LCM, ketogenic diet	Sultiame, VPA, VBG, PLP, Rufinamide, methy-prednisolone, ketogenic diet	PB
Outcome	Uncontrolled	Uncontrolled	Daily seizures	Uncontrol	Controlled by PB, isolated GTCS at 6 m with PB withdrawn, frequent absences without medication
EEG	Burst suppression evolving into hypsarrhythmia	Multifocal epileptic discharges evolving into generalized epileptic discharges	Persistently abnormal with a disorganized background, decrements, multifocal epileptic activity or hypsarrhythmia	Hypsarrhythmia evolving to intermittent sharp-slow waves and general slowing, current EEG showing bilateral spike waves with central maximum	Rhythmical activity which was initially observed over the left parieto-auricular region and right temporal area evolving into clear focal sharp wave discharges over the left temporo-parietal area
**Neurological features**					
Before seizure onset	–	Profound developmental delay at birth	Profound developmental delay at birth	Mildly motor developmental delay at birth	–
After seizure onset	–	Regression in development	Regression in development	Developmental arrest	Delayed milestones of development
Current development	Profound developmental delay, poor visual contact, head deviation to one side	Sever intellectual disability, no language, no verbal responses, walk with significant assistance	Truncal instability, limited visual attention, no verbal responses, walk with significant assistance	Sever intellectual disability, no language	Sever language delay and learning disability, aggressive and uncooperative
Imaging	Normal	Normal	A generalized reduction in white matter and thinning of the corpus callosum	Stable supratentorial mild volume loss and slightly delayed myelination	Normal
**Additional features**					
Dysmorphic features	Hirsute arms, thick hair, prominent eyebrows, and long and thick eyelashes, broad nasal tip, short and smooth philtrum	Hirsute arms, thick hair, prominent eyebrows, and long and thick eyelashes, broad nasal tip, short and smooth philtrum	No	Hirsute arms, thick hair, prominent eyebrows, and long and thick eyelashes, broad nasal tip, short and smooth philtrum with prominent upper lip, mild tooth displacement with diastema	Hirsute arms, thick hair, prominent eyebrows, and long and thick eyelashes, broad nasal tip, short and smooth philtrum without prominent upper lip, mild tooth displacement with diastema, and slightly long, spatulate fingers, with slightly deep-set nails
Other medical issues	No	No	No	No	No
Gender/Age	Female/3m	Female/29y	Male/6y	Male/5y	Male/17y
Variant	c.1690A>T, p.(Lys564*)	c.143-144del, p.(Leu48Glnfs*43)	c.544A>T, p.(Asn182Ile)	c.2638C>A, p.(Leu880Met)	c.725C>A, p.(Thr242Asn)
Transcript	NM_198503.2	NM_198503.2	NM_198503.2	NM_198503.4	NA
Functional analysis	Loss-of-function	Loss-of-function	NA	NA	NA
Diagnosis	EIMFS	DEE with migrating focal seizures (EIMFS-like)	DEE	DEE	DEE with clinical features of frontal lobe epilepsy
Age at epilepsy onset	2 m	4 m	Unknown	Unknown	5 m
Seizure type	Focal seizures	Focal and migrating seizures	Unknown	Unknown	Tonic motor seizures, hyperkinetic focal motor seizures
Antiepileptic Treatment	VPA, LTG, LEV	Unknown	Unknown	Unknown	CBZ, LEV, OXC
Outcome	Uncontrol	Uncontrol	Unknown	Unknown	Hyperkinetic focal motor seizures twice a month
EEG	Symmetric slow background pattern, multifocal spikes and seizures arising from different regions independently and migrating from one hemisphere to the other at time	Unknown	Sharp and slow waves in the right frontotemporal region	Normal at the age of 1 year old	Disorganized background, multifocal epileptiform discharges (predominantly frontocentral)
**Neurological features**					
Before seizure onset	Unknown	Unknown	Unknown	Unknown	Profound developmental delay at birth
After seizure onset	Unknown	Unknown	Regression in development	Regression in development	Regression in development
Current development	Sever neurologic impairment with the poor visual following	Mild intellectual disability	Delayed neural development	Delayed neural development	Sever intellectual disability with autistic features, mild spasticity, ataxic gait, partially dependent for daily life activities
Imaging	Normal	Unknown	Diffusely thin corpus callosum, dilated lateral ventricles and partial colpocephaly		Normal
**Additional features**					
Dysmorphic features	No	No	No	No	No
Other medical issues	No	No	Hypotonia	Hypotonia	No

*IS, infantile spasms; DEE, development and epileptic encephalopathy; EIMFS, the epilepsy of infancy with migrating focal seizures; LGS, Lennox-Gastaut syndrome; ES, epileptic spasms; EEG, electroencephalogram; PB, phenobarbitone; TPM, topiramate; VPA, valproic acid; LTG, lamotrigine; NZP, nitrazepam; LEV, levetiracetam; VGB, vigabatrin; ESX, ethosuximide; PLP, pyridoxal phosphate; LCM, lacosamide; GTCS, generalized tonic clonic seizure; CBZ, carbamazepine; OXC, oxcarbazepine*.

**Figure 1:**
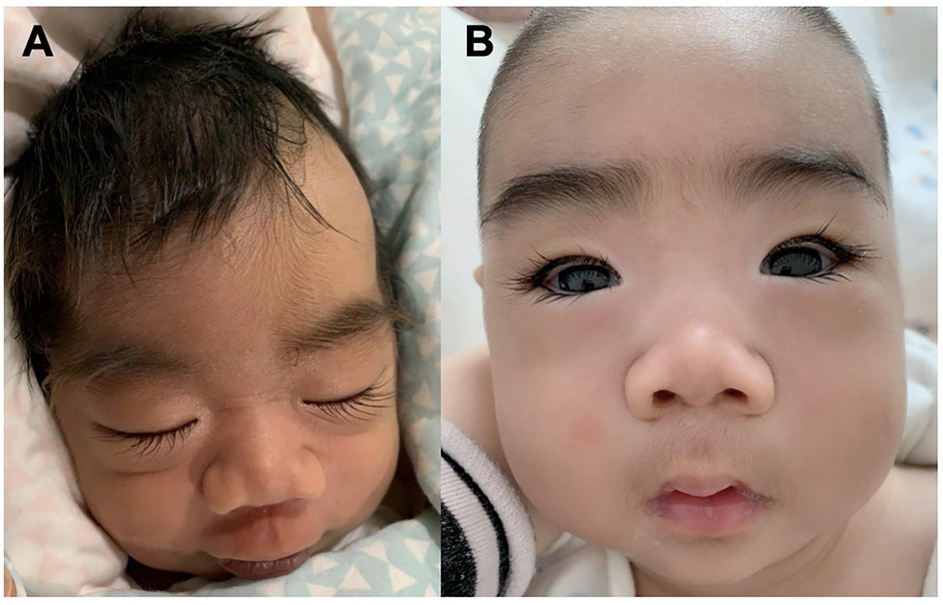
Facial Gestalt of the Patient #1 with *de novo KCNT2* variant **(A)** at birth and **(B)** at the age of 3 months.

Patient #1 was a 5 month-old boy. He was born at 38 weeks of gestation with a birth weight of 2,950 g. He was the first child of healthy non-consanguineous parents. There was no familial history of any neuropsychiatric disease including epilepsy or febrile seizures. He was hypotonic and had a profound delayed development. From 8 days of age, the boy presented with daily clusters of epileptic spasms. The EEG showed burst suppression. At the current age of 5 months, the boy was unable to hold his head with poor visual contact. His body measures were 66 cm and 6.7 kg. The seizures were still uncontrolled on the combination of phenobarbitone and topiramate at the last follow-up. During the course, the semiology evolved from Ohtahara syndrome to West syndrome with severe developmental delay and frequent epileptic spasms. EEG displayed with hypsarrhythmia. Brain magnetic resonance imaging (MRI) at 18 days was normal. The patients manifested dysmorphic features with hirsute arms, thick hair, prominent eyebrows, and long and thick eyelashes and had a broad nasal tip, short and smooth philtrum (Figure 1).

Patient #2 was a 9 year-old boy. He was born at full term of gestation with a birth weight of 3,300 g. He was the first child of healthy non-consanguineous parents. There was no familial history of any neuropsychiatric disease including epilepsy or febrile seizures. He had a profound delayed developmental milestone with raising his head at the age of 1 years and starting to walk at the age of 5 years. According to his parents and medical records, the boy presented with daily seizures of awaking from sleep with the head swinging from side to side and the limbs jerking at 45 days of age. Seizures were mainly focal and migrating. EEG showed multifocal epileptic discharges with left temporal predominance. At the current age of 9 years, the boy had severe intellectual disability, was able to walk with assistance, but had no language and no verbal response. His body measures were 118 cm and 20 kg. He still had frequent seizures under the combination of valproate, topiramate, and clonazepam. The current EEG displayed generalized discharges. Brain MRI at 4 years was normal.

**Figure 2:**
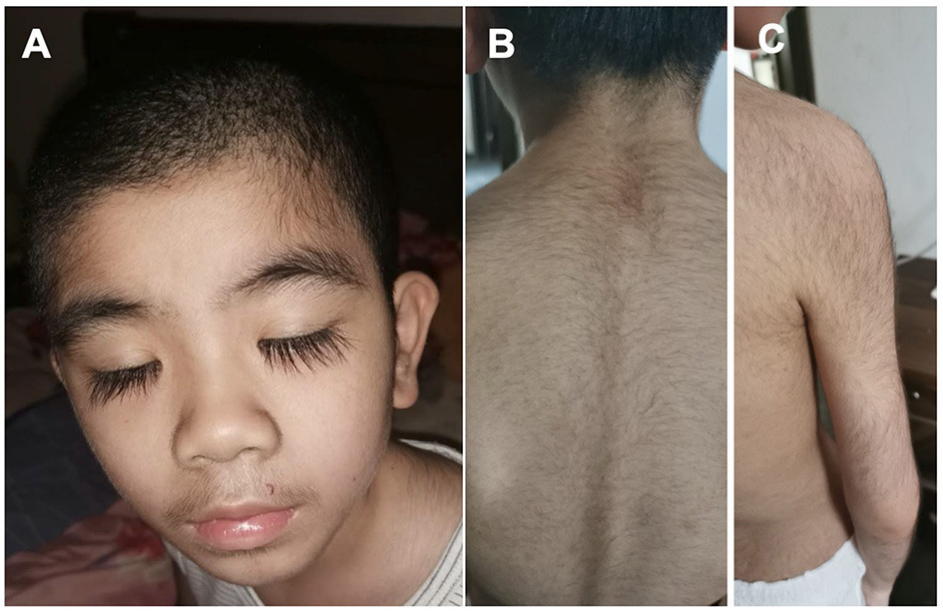
Dysmorphic features of the Patient #2 with *de novo KCNT2* variant at the age of 10 years. **(A)** Facial Gestalt of thick hair, prominent eyebrows, and long and thick eyelashes. **(B)** Thick hair on the back. **(C)** Thick hair on the right arm.

The patients manifested dysmorphic features with hirsute arms, thick hair, prominent eyebrows, and long and thick eyelashes and had a broad nasal tip, short and smooth philtrum (Figure 2).

## Genetic Analysis

**Figure 3:**
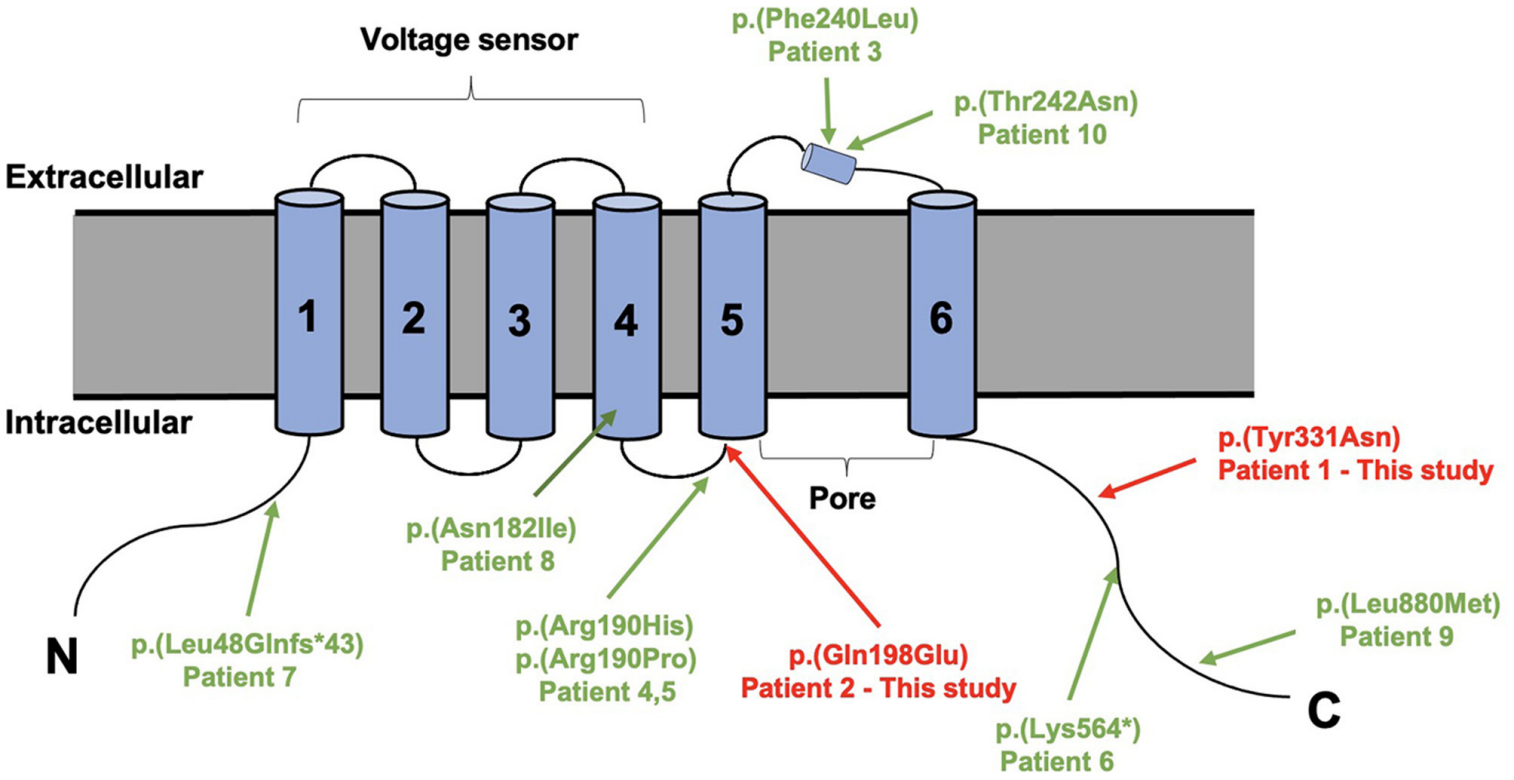
Structure of the sodium-activated potassium (K_Na_1.2) channel (9606 [NCBI]) with all variants published variants in *KCNT2*.

Trio-based whole-exome sequencing revealed *de novo* missense variants of [Chr1:196,395,112A>T, c.991T>A, p.(Tyr331Asn)] and [Chr1:196,465,339G>C, c.592C>G, p.(Gln198Glu)] in *KCNT2* gene (NM_198503.5, GRCh37/hg19), respectively, in two patients. Both variants were confirmed by conventional Sanger sequencing. The variants were novel and absent in control populational databases, including 1000 genomes, ExAC, and gnomAD. The pathogenicity of two novel variants was further analyzed using PolyPhen-2 (http://genetics.bwh.harvard.edu/pph2/). Y331N and Q198E substitutions were shown to be possibly damaging (score 0.619 and 0.598, respectively). MutationTaster program (http://www.mutationtaster.org/) showed that both variants were predicted to cause change in protein function and were disease causing. Y331N was localized in the C-terminal part of K_Na_1.2 and Q198E was localized in S4-S5 of the protein (Figure 3). Moreover, the phenotype of the patients was consistent with that of *KCNT2*-associated disease. Therefore, both variants were classified as likely pathogenic based on the American College of Medical Genetics (ACMG) guidelines, both mutations were classified as pathogenic with the PS2, PM2, and PP3 criteria (Richards et al., 2015).

## Discussion

As a recently discovered gene associated with epilepsy disorders, a total of eight patients bearing five *KCNT2* variants had been reported (Gururaj et al., 2017; Ambrosino et al., 2018; Alagoz et al., 2020; Inuzuka et al., 2020; Mao et al., 2020). The *KCNT2*-associated DEEs comprises West syndrome as well as epilepsy of infancy with migrating focal seizures (EIMFS). In 2017, Gururaj et al. firstly identified a *KCNT2* mutation in an early onset epileptic encephalopathy (EOEE) patient with intractable epileptic spasms by exome sequencing and confirmed the mutation with “change-of-function” effect (Gururaj et al., 2017). At the last follow-up age of 4 years, the patient presented with prolonged tonic seizures, myoclonic jerks and atypical absences with a disorganized background, decrements, multifocal epileptogenic activity or hypsarrhythmia on EEG (Gururaj et al., 2017). We assumed that the semiology of this patient was likely to evolved from infantile spasms (IS) to Lennox-Gastaut syndrome (LGS). In 2018, Ambrosino et al. described two female patients with IS followed by LGS or with DEE with migrating focal seizures. *In vitro* analysis further suggested that the variants found in patients had gain-of-function effects (Ambrosino et al., 2018). Seizure discharges in the patient with DEE with migrating focal seizures had been observed to migrate from the left to the right hemisphere, which likely corresponded to EIMFS (Ambrosino et al., 2018). Most recently, Mao et al. identified two novel *de novo* non-sense and frameshift mutations of the *KCNT2* gene in two patients diagnosed as EIMFS and early onset epileptic encephalopathy (EOEE) with migrating focal seizures, respectively (Mao et al., 2020). Due to the fact that the latter case was an aged case, diagnosis was not be firmly ascertained and the author described the patient as EIMFS-like EOEE (Mao et al., 2020). They investigated the functional consequence of the two mutations, which showed that both mutations reduced whole-cell potassium current (Mao et al., 2020). Subsequently, there were two reports describing three patients with EOEE caused by mutations in *KCNT2* without functional analysis (Alagoz et al., 2020; Inuzuka et al., 2020). Here, our patients showed DEE in common with previous studies. Patient #1 was diagnosed as Ohtahara syndrome subsequently followed by IS with EEG showing burst suppression evolving to hypsarrhythmia. Patient #2 was diagnosed as DEE with migrating focal seizures manifesting profound developmental delay at birth and intractable seizure with developmental regression. Due to incomplete medical records, there was no definitive diagnosis but EIMFS-like EOEE of Patient #2. In general, both gain and loss of function mutations in the *KCNT2* gene could lead to DEE, and the most common phenotypes were IS and EIMFS. The epilepsy phenotype associated with gain- and loss-of-function mutations could overlap.

EIMFS is a rare EOEE characterized by polymorphous focal seizures and cognitive, sensory and motor impairment, with arrest of psychomotor development in the first 6 months of life (Coppola et al., 1995, 2006). EIMFS have a genetic origin in which the *de novo* gain-of-function *KCNT1* variants are the most common cause (Barcia et al., 2012; McTague et al., 2013; Rizzo et al., 2016; Villa and Combi, 2016). *KCNT1* and *KCNT2* respectively encode the K_Na_1.1 (Slack) and K_Na_1.2 (Slick) subunits. Both genes belongs to the SLO2 family of the sodium-dependent voltage-gated potassium channel K_Na_ (Bhattacharjee et al., 2003). Functional analysis of mutant channels associated with EIMFS mostly revealed gain-of-function effects (Barcia et al., 2012; Rizzo et al., 2016; Villa and Combi, 2016). Similarly, Ambrosino et al. identified a gain-of-function mutation in the *KCNT2* gene in a patient with EIMFS-like DEE. It suggested that pathogenic variants in *KCNT1* and *KCNT2* therefore might contribute to a similar and overlapping spectrum of DEEs (Ambrosino et al., 2018; Kessi et al., 2020). Meantime, Mao et al. also reported EIMFS in patients carrying loss-of-function mutations in the *KCNT2* gene (Mao et al., 2020), which indicated that EIMFS might be caused not only by an increase but also by a decrease in the function of K_Na_.

Moreover, it was noticed that the two patients confirmed to carry gain-of-function mutations both exhibited not only severe developmental delay, but also dysmorphic features with hirsute arms, thick hair, prominent eyebrows, and long and thick eyelashes, broad nasal tip, short and smooth philtrum with prominent upper lip, mild tooth displacement with diastema (Ambrosino et al., 2018). Similarly, both of our patients had prominent dysmorphic features with hirsute arms, thick hair, prominent eyebrows, and long and thick eyelashes. We presumed that the dysmorphic features might be the distinguishing characterizations in patients with gain-of-function mutations in *KCNT2*. However, we could not confirm the mutations in our patients in the absence of functional analysis. Given the small number of patients reported so far, it is difficult to make genotype-phenotype correlations. Additional *KCNT2* cases will help clarify the potential association between gain-of-function and dysmorphic features.

Seizures in all patients were intractable and there was a poor prognosis in development. The oldest patient at the age of 29 years still had seizures and showed mild intellectual disability (Mao et al., 2020). Quinidine as an underlying precision medicine for epilepsy syndromes due to gain of function mutations in *KCNT1* aroused particular clinical interest (Bearden et al., 2014). Therefore, Ambrosino et al. confirmed by functional analysis that the patients carried quinidine-responsive gain-of-function mutations in the *KCNT2* gene and treated one patient with quinidine add-on therapy and achieved marked clinical improvements, including EEG, vigilance, alertness as well as developmental progression (Ambrosino et al., 2018). It suggested that quinidine could be a potential personalized medicine approach for *KCNT2*-related DEE.

## Conclusion

In this study, we presented the detailed clinical features and genetic analysis of two unrelated patients with *KCNT2*-related DEE and provided a comprehensive outline of available publications regarding *KCNT2* mutations. Our data further expanded the spectrum of *KCNT2* mutation. So far, with our case, a total of ten cases carrying *KCNT2* mutations have been reported. IS and EIMFS were the most common phenotypes caused by pathogenic mutations in *KCNT2*. Both gain and loss of function mutations could lead to EIMFS. Additional *KCNT2* cases will help to make genotype-phenotype correlations clearer. Quinidine could be a potential personalized medicine approach for *KCNT2*-related DEE. Clinical follow-up of additional patients will further define the clinical spectrum of *KCNT2*-related DEE and the long-time efficacy on seizures and development of quinidine as an underlying precision medicine for epilepsy syndromes due to gain-of-function mutations.

## Data Availability Statement

The sequencing data are available on https://www.ncbi.nlm.nih.gov/bioproject/PRJNA689060.

## Ethics Statement

The studies involving human participants were reviewed and approved by the Ethical Committee of Peking University First Hospital. Written informed consent to participate in this study was provided by the participants' legal guardian/next of kin. Written informed consent was obtained from the minor(s)' legal guardian/next of kin for the publication of any potentially identifiable images or data included in this article.

## Author Contributions

ZY conceptualized and designed the study, coordinated the study overall, and revised the manuscript. PG co-designed the study, drafted the initial manuscript, and revised the manuscript. XJ and DY helped to collect and summarize data and revised the manuscript. All authors approved the final revision of the article.

## Conflict of Interest

The authors declare that the research was conducted in the absence of any commercial or financial relationships that could be construed as a potential conflict of interest.
